# Heart and liver are infected in fatal cases of dengue: three PCR based case studies

**DOI:** 10.1186/s12879-018-3603-x

**Published:** 2018-12-19

**Authors:** S. A. M. Kularatne, M. M. Rajapakse, Udaya Ralapanawa, R. Waduge, L. P. M. M. K. Pathirage, R. P. V. J. Rajapakse

**Affiliations:** 1 Department of Medicine, Faculty of Medicine, University of Peradeniya, Peradeniya, Sri Lanka; 2 Department of Pathology, Faculty of Medicine, University of Peradeniya, Peradeniya, Sri Lanka; 3 Department of Veterinary Pathobiology, Faculty of Veterinary Medicine and Animal Science, University of Peradeniya, Peradeniya, Sri Lanka

**Keywords:** Dengue, Myocarditis, Liver failure, Expanded dengue syndrome, RT-PCR, Sri Lanka

## Abstract

**Background:**

Dengue is a global problem mainly in the tropics. Meticulous clinical management of cases has reduced the death rate significantly, but large numbers of people still succumb to severe complications of the infection. Presence of myocarditis is often overlooked leading to a poor outcome. Clinical management guidelines of dengue do not stress the importance of myocarditis as a manifestation in dengue infection. Severe hepatic dysfunction also needs emphasis.

**Case presentation:**

We present three patients who had come to hospital on the 3rd day of fever. Two of them (case 1 and 3) were in shock on admission and case 2, who was stable on the3^rd^ day, went into the critical phase and developed shock while in the hospital on the 4^th^day. All three had tachycardia on admission that got worse with time. The clinical course was unstable with fluctuations in urine output and deterioration of organ function. Despite frequent monitoring and life support they survived only 2–3 days in hospital. All three patients had myocarditis during the critical phase. In the first case, myocarditis was confirmed by troponin estimation and echocardiogram. In the second and third cases, histopathology confirmed myocarditis. Haemorrhagic necrosis of the liver was found in case 2 and 3 with exponential rise of transaminases. In all three cases, viral RNA was detected in both heart and liver tissues by PCR amplification.

**Conclusions:**

We stress that detection of myocarditis and liver involvement in any dengue patient is important from the onset of the illness where treatment should be tailored to prevent development of hypotension. Our findings are novel as PCR and histology are rarely done on tissues of deceased dengue patients in the world. Studies are needed to find therapeutic interventions to reverse cardiac and hepatic dysfunction in dengue infection.

## Background

Dengue is a global problem, mainly in the tropics, and travelers visiting endemic regions are at risk of contracting the infection [1–3]. Meticulous clinical management of cases has reduced the death rate significantly, but large numbers of people succumb to severe complications of the infection [4]. Sri Lanka experienced the worst dengue epidemic in mid-2017 with a total case load of the year amounting to 185,000 cases, with more than 400 deaths [5]. At institution level and centrally at the Ministry of Health, these unfortunate deaths are being reviewed in order to improve case management and to understand the contributory factors responsible for death. However, despite improvement of necessary infrastructure and guideline-based management, dengue infection still causes deaths [6]. The main pathology of dengue haemorrhagic fever is plasma leak from capillaries, where all kinds of cytokines are involved,but is amenable to meticulous fluid management [4, 6]. Moreover, WHO recognizes the expanded dengue syndrome to explain uncommon complications of the infection [7]. The majority of patients recover from the infection, if plasma leak is the sole pathology. Such a notion in the clinician’s mind tends to overlook the concomitant vital organ dysfunction that is present in some cases of dengue. In these three case studies, we aim to demonstrate the presence of virus in the organ tissue at necropsy examination and to collate those findings with the clinical and laboratory manifestations prior to death to show evidence of dengue infection in the heart and lungs.

## Case presentation

### Case 1

A 34-year-old male presented to the THP on 11th May 2017 with a 3-day history of fever, arthralgia, backache, headache and skin flush. He had no cough, abdominal pain or diarrhoea. On admission, he was ill looking and had postural giddiness and cold peripheries. Blood pressure was 80/50 mmHg and pulse rate was 98 beats/min. Resuscitation was attempted with rapid infusion of 500 ml of normal saline followed by continuation normal saline infusion. His blood pressure (BP) picked up to 100/70 mmHg, but he remained oliguric over the next 12 h. Dextran 40 colloid 500 ml bolus infusion was given to raise the BP and to produce more urine. Twelve hours later, he developed generalized convulsions and needed immediate intubation and assisted ventilation in the Intensive Care Unit (ICU). Chest radiograph showed bilateral lung shadows suggestive of pulmonary oedema. Other investigations are shown in Table 1. In the ICU, at 3 PM, BP was 130/80 mmHg and pulse rate was 130 beats/min. However, at 3.30 PM, pulse rate was 160 beats /min, and BP dropped to 97/53 mmHg. The patient had central cyanosis, cold peripheries, feeble peripheral pulses and the haematocrit (HCT) rose to 55% requiring more Dextran 40 infusion.

**Table 1:** Summary of important clinical and laboratory data of 3 fatal cases of dengue with the list of complications

Description of case		Clinical status on admission and subsequently	Investigations		Complications	Duration of illness from onset to death
No	Age/Gender		Test	Results		
1	34 / M	Fever 3 days	WBC	3.5 × 10^9^/l	Persistent hypotension	5 days
		Postural giddiness	Platelets	28 × 10^9^/l	Fluid overload	
		Cold peripheries	Creatinine	193 μmol/l	Pulmonary oedema	
		BP: 80/50 mmHg	CRP	320 mg/l	Left ventricular-	
		Pulse rate 98/min	ALT	73 U/L	hypokinesia	
		Convulsions	AST	98 U/L	Hypoxaemia	
		Assisted ventilation	Troponin I	0.29 ng/ml	Sepsis	
			PH	7.1	Lactic acidosis	
			Pco2	44 mmHg	Bleeding	
			PaO2	61 mmHg	Renal failure	
2	36/M	Type 2 diabetes	WBC	3.7 × 10^9^/l	Erratic critical phase	6 days
		Fever 3 days	Platelets	34 × 10^9^/l	Undue tachycardia	
		BP 130/80 mmHg	Creatinine	82.5 μmol/l	Hypotension	
		Pulse rate 90/min	Blood sugar	233 mg/dl	Fulminant hepatic	
		Critical phase on	ALT (day- 3)	383 U/L	failure	
		4th day with pleural effusion, tachycardia and low BP 100 mmHg Melaena, Reduced urine out put	(day-4)	4905 U/L	GI- bleeding	
			AST (day-3)	463 U/L	Acute kidney injury	
			(day-4)	9371 U/L	Lactic acidosis	
			INR (day-5)	> 5		
			PH (day-5)	7.32		
3	40/F	Bronchial asthma	WBC	8.2 × 10^9^/l	Profound shock on	6 days
		Fever 4 days	Platelet	28 × 10^9^/l	admission	
		Vomiting, shock	HCt	37	Pleural effusions	
		Cold peripheries	Creatinine	84 μmol/l	Fulminant hepatic	
		BP – un-recordable	Albumin	30.8 g/l	failure	
		Pulse-108/ min	ALT(day-4)	1365 U/L	Encephalopathy	
		US-Ab: fluid leak	(day-7)	2590 U/L		
		Abdominal pain	AST (day-4)	2999 U/L		
		Melaena Confusion	(day-5)	6812 U/L		

On morning of the fifth day at 5 AM, HCT and haemoglobin dropped to 28% and 9 g/dl respectively without any obvious bleeding. With transfusion of one unit of blood, BP rose to 130/95 mmHg. With further transfusion HCT and urine output improved. Two dimensional echocardiogram showed global left ventricular hypokinesia with an ejection fraction of 40%. Cardiac troponintitre was high. From noon, the BP was falling - 80/50 mmHg and tachycardia persisted that needed continuous inotrope infusion. Despite meticulous management, the patient developed cardiac arrest and resuscitation failed.

### Case 2

A 36-year-old male suffering from type 2 diabetes for 3 years was transferred to the Teaching Hospital, Kandy on the 15th July, 2017 with a 3-day history of fever and arthralgia.On admission he was febrile and BP was 130/80 and pulse rate was 90 beats per min. Lungs were clear and abdomen was soft with no hepatomegaly. Initial investigations revealed WBC of 3 × 10^9^/l, platelets of 63 × 10^9^/l and HCT of 45%. On day 1 of admission, his basic clinical parameters were normal and urine output was adequate throughout the day. On the next day, ultrasound scan examination of abdomen showed evidence of early plasma leaking, thus initiating critical phase monitoring from 9 am on 16th July. At this point his BP was 100/70 mmHg and HCT was 45%. His WBC was 3.7 × 10^9^/l, platelet count was 34 × 10^9^/l. His alanine transaminase (ALT) and aspartate transaminase (AST) were 383 U/l and 463 U/l respectively.

From the onset of the critical phase, the patient had tachycardia which persisted. He had satisfactory urine output initially, but his HCT continued to increase. At about 7 pm, a Dextran-40 colloid 500 ml bolus was given, but at midnight the urine output started dropping to a level of 35 ml/h. Thus, another bolus of 250 ml of dextran-40 was given. Meanwhile, the patient developed melaena, and 500 ml of whole blood was transfused. As the patient developed lactic acidosis, sodium bicarbonate infusion was given.

The patient was transferred to the ICU, Teaching hospital, Peradeniya (THP) at 3 am of 17th July while managing the critical phase into the 18th hour. On admission, blood pressure was 125/91 mmHg which rapidly dropped to 100/80 mmHg and the peripheries were cold to touch. The patient was conscious and rational. The urine output dropped to 20 ml/h. Pulse rate remained at 140 beats/min. Auscultation of the lungs revealed reduced breath sounds with fine crepitations in both lungs. Melaena was present on rectal examination. Arterial blood gasses showed lactic acidosis. Ultrasound examination of abdomen showed a significant amount of ascites. The patient was given two 250 ml boluses of dextran-40. The investigations are shown in Table 1. By about 7 AM, the patient became anuric with BP of 99/65 mmHg and HCT was 50%. The patient was transfused with 3 units of blood within the next 2 h but he remained anuric. By 9 AM, the patient was confused and disoriented and had cold peripheries with sluggish capillary refilling. He was intubated and ventilated electively. At 1.20 pm the patient developed bradycardia followed by cardiac arrest and resuscitation was unsuccessful.

### Case 3

A 40-year-old female with a history of bronchial asthma for 17 years, presented to the THP with fever of 3 days duration associated with vomiting and postural giddiness on 22nd July 2017. On admission, pulse rate was 108 beats /min and blood pressure was unrecordable. Ultrasound scan of the abdomen showed evidence of fluid leaking. The onset of critical phase was calculated 8 h backward and prompt resuscitation was attempted. Despite fluid resuscitation tachycardia persisted with reduced urine output. On admission her ALT was 1365 U/l and AST was 2999 U/l. Serum albumin was 30.8 mg/dl and serum creatinine was 84 μmol/l. Her INR was 1.38. Serum amylase was 37 U/L.

On the evening of the same day she complained of abdominal pain with tenderness of the right hypochondrium. She passed melaena stools. She had tachypnoea and blood gasses revealed acidosis. A small right sided pleural effusion was also present. She was transfused with one unit of whole blood. By the next morning her abdominal pain and postural symptoms increased and she developed mild icterus. She had tachycardia, but the blood pressure was maintained and urine output was satisfactory. She then developed ascites and bilateral pleural effusions. The next AST was 3661 U/l and ALT was 1579 U/l. By the evening her oxygen saturation dropped despite administering 100% oxygen. Subsequent AST and ALT were 8543 U/l and 2981 U/l respectively. The serum amylase was 48 U/l and serum creatinine was 105 μmol/l.

Towards the latter part of the critical phase the HCT started falling and the urine output declined to 25 ml/h. Several units of blood, FFP, and human albumin were infused to maintain the HCT. The patient rapidly developed difficulty in breathing with bilateral moderate pleural effusions. However, her AST and ALT levels reduced to 3661 U/l and 2685 U/l. On 24th July, the critical phase monitoring was over, but the patient gradually became confused and disoriented. She had high fever and oral bleeding but her other vital parameters and urine output were satisfactory. There were crepitations all over the lung fields and she needed assisted ventilation in the ICU. She died on the 2nd day in the ICU.

## Discussion and conclusions

**Fig. 1:**
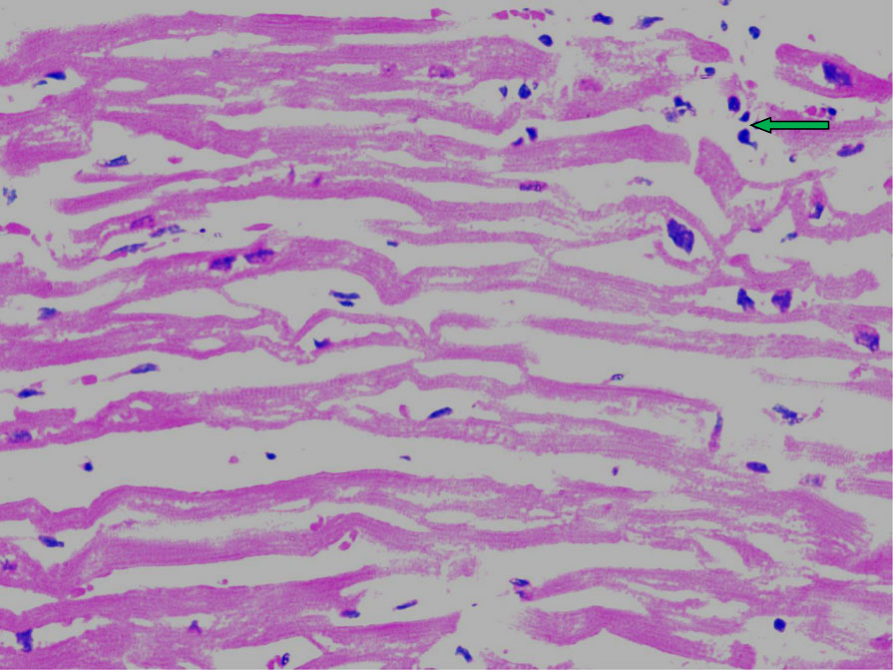
Heart muscle × 400 inflammatory cell infiltration shown in arrow

**Fig. 2:**
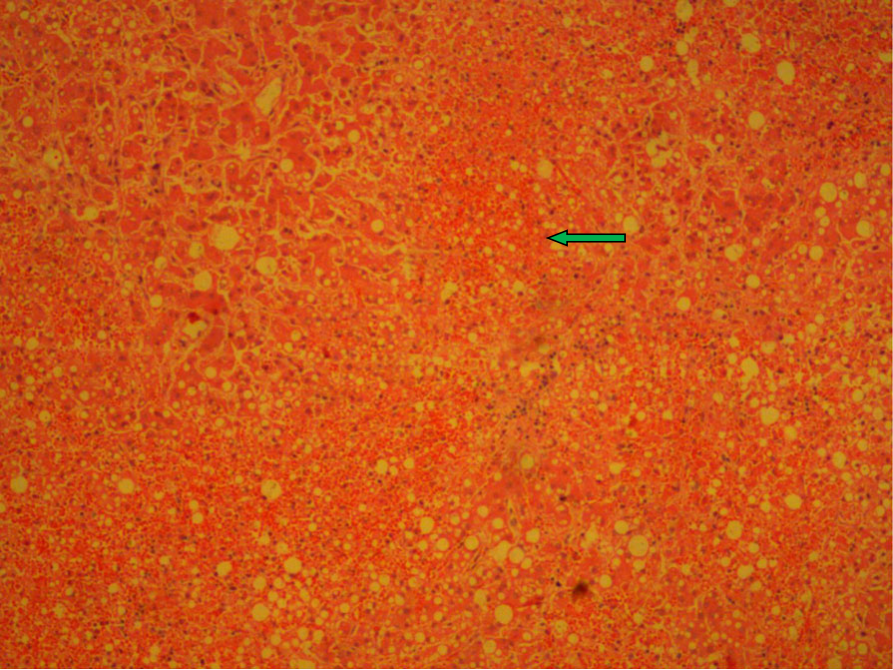
Liver × 100 bringing necrosis shown in arrow

All three patients entered hospital on the 3rd day of fever.Two of them,cases 1 and 3, were in shock on admission and case 2, who was stable on admission on the 3rd day of fever,went into critical phase and shock while in the hospital on the 4th day. All three had tachycardia on admission that increased with time. The clinical course was unstable with fluctuations in urine output and deterioration of organ function. With very close monitoring and intense life support they lived only 2–3 days in the hospital. All three patients had myocarditis complicating the critical phase. In the first case, myocarditis was confirmed by cardiac troponin estimation and echocardiogram. In the second and third cases, post mortem histopathology confirmed myocarditis (Table 2, Fig. 1).PCR amplification was used to demonstrate the presence of dengue viral RNA in the myocardium in all three cases. The liver involvement is more marked in case 3 as evident by very high levels of hepatic transaminases. Case 2 and 3 had extensive haemorrhagic necrosis of the liver (Table 2, Fig. 2). In all three cases, viral RNA was detected by PCR in the liver. Moreover, viral RNA was detected in many tissues of these patients.

**Table 2:** Detection of Dengue virus RNA in different tissue samples obtained at necropsy examination and respective gross and microscopic changes in tissues of fatal cases of dengue

Tissue	Case 1		Case 2		Case 3	
	PCR	Macroscopy	PCR	Histology	PCR	Histology
Brain	–		+	Normal	–	Normal
Lungs	+	Congested, Haemorrhagic	*	Congested capillaries	+	Pulmonary oedema, capillary congestion in interstitium
Heart	+	Petechial haemorrages	+	Interstitial oedema with infiltration of neutrophils and lymphocytes	+	Marked interstitial oedema, lymphocytic infiltration in endomyseum
Liver	+	Congested	+	Extensive haemorrhagic necrosis	+	Submassive necrosis, haemorrhagic necrosis
Kidney	+		+	Acute tubular necrosis	–	Acute tubular necrosis
Spleen	–		–	Expansion of red pulp, extravasations of red cells	–	Expansion of red pulp with haemorrhage
Adrenal gland	*		*		+	Early autolysis
Pancreas	*		*		+	Early autolysis
Intestines	+		*			
Muscles	*		+	Occasional fibre necrosis, inflammation	+	
Skin	+		*		*	
Blood	+		*		*	

(+) detection of dengue (DEN 2) RNA in tissues, (−-) Dengue RNA not detected, (*) Not tested as tissues samples were not available. In Case 1, histology of tissues were not done

Postmortem studies are seldom reported in the world despite many dengue deaths. Further, histology and PCR studies of tissues of deceased victims are hardly happening despite availability of technology. Therefore, organ involvement in dengue infection has not been fully understood. This study shows extent of organ involvement in dengue and how it collates with the clinical manifestations in severe cases of dengue. There is a possibility that dengue virus infected blood cells entrapped in the organs would have given positive PCR results. However, taking clinical evidence of organ dysfunction, biochemical derangements and histology together, it is most likely that dengue virus could infect the organs.

Literature to support the existence of myocarditis in dengue infection has been available for more than a decade [8–15]. However, clinical management guidelines have given only little importance to myocarditis as a manifestation in dengue infection [6, 15]. In 2005, clinical experience in managing a large series of dengue cases with myocarditis in Sri Lanka was published where the causative serotype was DEN 3 [9, 13, 14]. Histopathological evidence of myocarditis was also found in 2009 [13]. A traveler returning from the Dominican Republic developing myocarditis has been published in 2014 [8]. Even though, there have been many deaths due to dengue, the literature available on necropsy studies dealing with detection of virus in tissues and related histopathology is scarce [16–21]. This deficit has curtailed critical analysis and identification of the true contributory causes for deaths. Clinicians are trapped in a straitjacket of orthodox thinking of clinical management of dengue where some patients die due to irreversible shock. It has been observed that fluid overload has contributed to most dengue deaths, like in case 1 in this series. In pure fluid leaking DHF, fluid overload does not occur if the cardiac status is normal. However, if myocarditis is present, the heart naturally fails when fluid is given in excess. The presence of tachycardia and development of shock early in the disease should sound the alarm of myocarditis. To prevent deaths, early detection of myocarditis is essential to institute extra precautions needed in management [9, 13, 14].

Hepatic involvement in dengue has been addressed in guidelines and literature [6, 7, 22, 23]. However, hepatic involvement has been ascertained by rising transaminases and in extreme cases development of fulminant hepatic failure. Very often, the pathophysiology that leads to hepatic dysfunction remains unknown. Speculation of ischemic hepatic damage during the critical phase is often entertained. In 2014, we demonstrated extensive haemorrhagic necrosis of liver in a necropsy study along with detection of viral RNA in liver tissue [23]. In the present case series, we found similar findings. In general, all cases of dengue infection have elevated transaminases at different levels [7, 24–26]. In some cases, elevation of transaminases is very high [24]. This suggests that the dengue virus infects the liver in virtually all cases, rarely causing severe hepatitis. Minimizing liver damage in dengue is a problem that needs resolving: some clinicians use N acetyl cystine infusions on a regular basis when hepatic transaminases are high [27, 28]. Studying these three cases suggests that the dengue virus could infect any tissue in the body even skin, muscles and intestine (Table 2). This is usually reflected in the clinical manifestations of dengue e.g. muscle pain, skin rashes, abdominal pain, diarrhoea etc. It is of interest that in this study viral RNA was not detected in the spleen. Clinically, these three cases demonstrate multiple organ dysfunction, metabolic abnormalities, sepsis and bleeding along with persistent hypotension contributing to death. Once the balance of critical parameters tilts towards deterioration, it becomes irreversible and death becomes inevitable despite giving the best of care and life support. We can extrapolate our findings to explain most of the fatalities in dengue in Sri Lanka and even the world over. Therefore, we stress that detection of myocarditis and liver involvement in any dengue patient is important from the onset of the illness where treatment should be tailored to prevent development of hypotension. Studies are needed to find therapeutic interventions to revert cardiac and hepatic dysfunction in dengue infections.

## Abbreviations

ALT: Alanine transaminase
AST: Aspartate transaminase
CRP: Creactive protein
HCT: Haematocrit
ICU: Intensive Care Unit
PCR: Polymerase chain reaction
WBC: White blood cells
